# Supplementary material for: Characterizing cell-type spatial relationships across length scales in spatially resolved omics data
Source: Nat Commun. 2025 Jan 3;16:350. doi: 10.1038/s41467-024-55700-1 (PMC11699133; doi:10.1038/s41467-024-55700-1)
Supplement: Supplementary file 3 — Supplementary Software 1 [file 41467_2024_55700_MOESM3_ESM.zip › CRAWDAD/plots/supplementary/S7_cluster_exp_FSLD_annot.pdf]

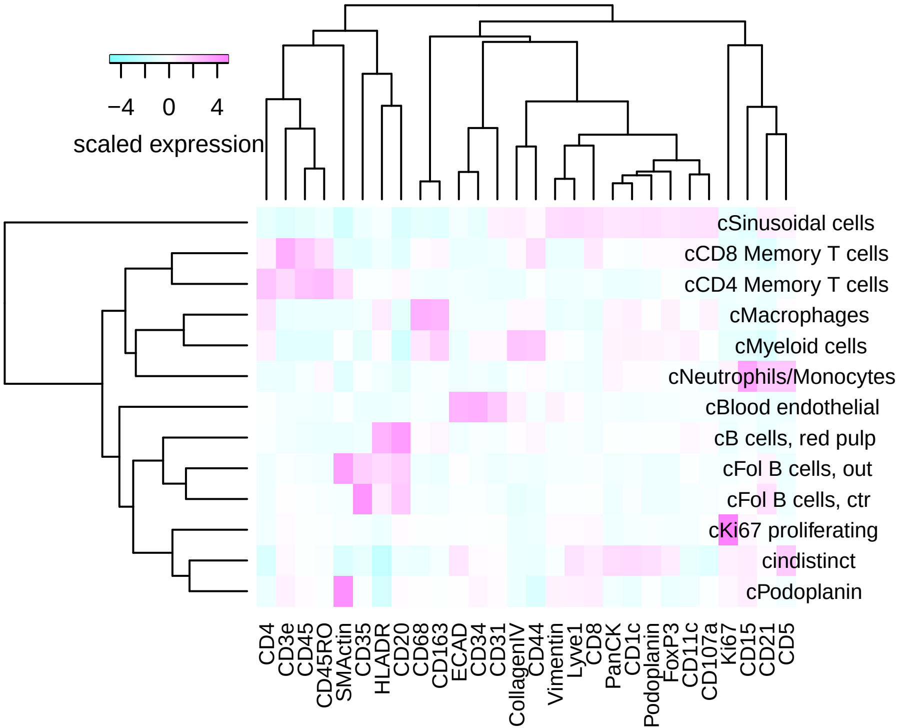

−4
0
4
scaled expression
cSinusoidal cells
cCD8 Memory T cells
cCD4 Memory T cells
cMacrophages
cMyeloid cells
cNeutrophils/Monocytes
cBlood endothelial
cB cells, red pulp
cFol B cells, out
cFol B cells, ctr
cKi67 proliferating
cindistinct
cPodoplanin
CD4
CD3e
CD45
CD45RO
SMActin
CD35
HLADR
CD20
CD68
CD163
ECAD
CD34
CD31
CollagenIV
CD44
Vimentin
Lyve1
CD8
PanCK
CD1c
Podoplanin
FoxP3
CD11c
CD107a
Ki67
CD15
CD21
CD5
